# How well do we know green gentrification? A systematic review of the methods

**DOI:** 10.1177/03091325221104478

**Published:** 2022-06-01

**Authors:** Jessica Quinton, Lorien Nesbitt, Daniel Sax

**Affiliations:** Department of Forest Resources Management, 98686 Faculty of Forestry, Vancouver, BC, Canada; Department of Forest Resources Management, 98686 Faculty of Forestry, Vancouver, BC, Canada; Department of Forest Resources Management, 98686 Faculty of Forestry, Vancouver, BC, Canada

**Keywords:** urban planning, environmental gentrification, gentrification, green space, urban forest, environmental justice, ecological gentrification

## Abstract

This systematic literature review identifies and critiques methodological trends in green gentrification research (focusing on studies of vegetative greening) and provides suggestions for advancing this field. Findings reveal (1) research has largely focused on U.S. case studies; (2) early work employed qualitative methods but quantitative analyses have become more common; (3) little attention has been paid to the influence of greening characteristics/functions and non-greening factors on gentrification; (4) the mechanisms through which greening leads to gentrification are not well understood, particularly on the demand side; and (5) despite being the main concern of green gentrification, displacement has not been well-documented.

## I Introduction

Urban greening is prominent in city planning agendas worldwide and is ascribed a diverse set of names, including urban forests, green space, natured-based solutions, and green infrastructure (Escobedo et al., 2019). These terms are defined differently depending on the context and users but broadly refer to natural/semi-natural areas within cities that provide benefits to humans (Taylor and Hochuli, 2017). Such benefits are often referred to as ecosystem services (Duinker et al., 2015; Lee and Maheswaran, 2010; Nesbitt et al., 2017) and are increasingly relevant in the advent of more frequent and severe climate-change impacts. However, equity and justice issues in the distribution of urban green space (Rigolon, 2016) and the procedural dimensions of their planning and management (Heynen et al., 2006; Buijs et al., 2016), can result in less greening and fewer benefits for marginalized communities—although such findings depend on measurement scale, geographical context, and greening characteristics (Boone et al., 2009; Baró et al., 2019). An emerging justice issue has been green/environmental/ecological gentrification, which is concerned with displacement, exclusion, or marginalization of residents in areas surrounding sustainable/green urban (re)developments as they attract wealthier in-movers (Gould and Lewis, 2017). The following subsections provide a brief overview of how green-gentrification research has emerged from the fields of gentrification, environmental justice, and political ecology. The rest of the paper uses a systematic review of green-gentrification literature to achieve the following objectives: (1) highlight trends in the methods used to study vegetative green gentrification; (2) determine whether the methods used have been sufficient for understanding gentrification and the role of greening; and (3) provide methodological suggestions and considerations for future research. While there have been several useful reviews on segments of green gentrification (Pearsall and Anguelovski, 2016; Cole et al., 2017; Angelo, 2019; Taki et al., 2021), only one has examined trends and suggested future research pathways for the field as a whole (Anguelovski et al., 2019). Our review extends and complements this latter work by providing a systematic look at how green gentrification has been studied and describing a framework for conceptualizing gentrification and the role of greening within it. It also shows the progress made in this rapidly expanding field in the past few years.

### 1 Gentrification

Gentrification was first used to describe the displacement of working-class individuals by the middle class (the “gentry”) and the associated changes in social character in some London neighborhoods (Glass, 1964). The concept has been applied in diverse contexts, and decades of research has produced a range of definitions and understandings. There have been calls for a broader, more “elastic” definition of gentrification (Clark, 2004; Davidson and Lees, 2005), while some criticize the definition as too broad and without meaning (Maloutas, 2012). The following four characteristics of gentrification from Davidson and Lees (2005) provide a usefully broad understanding: (1) capital reinvestment; (2) social upgrading by high-income in-movers; (3) landscape change; and (4) displacement^1^ of low-income groups. An early supply-side explanation of gentrification came from rent-gap theory (Smith, 1979) which posited that landlords seek to maximize profits by minimizing reinvestment in infrastructure, eventually creating a gap between capitalized and potential ground rent. This attracts investors to purchase and redevelop property to be marketed towards higher-income households. The demand-side theory that emerged in response asserted it was customer preference for such properties, resulting from increases in white-collar work and dissatisfaction with suburbia, driving gentrification (Ley, 1981). Supply and demand arguments have both been critiqued, but scholars largely see the need for both to understand gentrification (Lees et al., 2008), highlighting the need to employ methods that capture how and by whom gentrified landscapes are both produced and consumed—acknowledging the role of structures/institutions *and* individual agency (Hamnett, 1991). A myriad of subfields such as rural (Smith and Phillips, 2001), super- (Lees, 2003), new-build (Davidson and Lees, 2005), commercial (Zukin and Kosta, 2004), and tourism gentrification (Gotham, 2013) have emerged and question earlier assumptions about processes and outcomes of gentrification. All of these “mutations” of gentrification involve socioeconomic and cultural transformations due to (re)colonization by an upper class (Lees et al., 2008).

Education and occupation (and thus, class) have been central to gentrification research and theory, but other factors—and their interaction with class—such as gender, sexuality, and particularly ethnicity/race have been increasingly implicated (Lees et al., 2008; Lees and Phillips, 2018). Neoliberalism has also been increasingly discussed in relation to how state deregulation, dismantling of the welfare state, and the global scale of capital and cultural exchange influence gentrification (Smith, 2002), often in locally dependent ways (Lees et al., 2008). Despite expansion of gentrification research to broader geographical contexts, some question whether gentrification is a truly global concept (Bernt, 2016). Some earlier theories have been adapted to this increasingly global view, but researchers highlight the need to avoid conceptualizing gentrification as migrating from the North/West to South/East (Lees, 2012, 2018; Maloutas, 2012).

### 2 Environmental Justice and Political Ecology

Environmental justice (EJ) research has historically been linked with activism (Taylor, 2000), and the first reports linking racial and socioeconomic disparities to the siting of toxic-waste facilities emerged following community protests in the United States (United States General Accounting Office, 1983; Chavis and Lee, 1987). *Environmental racism* was coined to describe racial discrimination from environmental decision-making and the influence of race on the distribution of hazardous-waste facilities (Chavis and Lee, 1987). Conceptions of EJ differ between users and applications, but it broadly refers to fair and equitable distribution of environmental goods and bads and participation and recognition in environmental decision-making and governance. In this context, “environment” refers to places humans live, work, and play—not just areas of wilderness (Novotny, 2000). EJ does not refer to single detrimental impacts but complex histories of interactions between politics, society, and economy, all of which underlie injustice (Pellow, 2000).

Early EJ research followed a Rawlsian approach primarily concerned with distribution (Rawls, 1999). Quantitative spatial analyses were—and still are—common and highlighted the proximity of marginalized groups to environmental hazards (Holifield et al., 2018). Such research focused on issues of race and class, debating their relative influence (Brulle and Pellow, 2006). While early work focused on toxic-waste exposure, it expanded to issues of unsustainable industrialization, resource depletion, food systems, energy consumption, and climate change (Agyeman et al., 2016; Holifield et al., 2018). More recently, spatial analysis of access to environmental amenities, such as urban green spaces, have permeated the literature (Rigolon, 2016). The EJ framework has also been expanded by integrating normative theories of justice (Young, 1990). Under radical EJ, there are three main dimensions: distribution, procedure/representation, and recognition (Schlosberg, 2004). Justice as recognition and procedure were conceptualized to recognize and respect the membership and participation of individuals within a community and address the role of institutional processes/procedures in equitable decision-making and implementation (Young, 1990; Schlosberg, 2004).

Critical and radical EJ research has been more aligned with political ecology (PE), typically expanding beyond quantitative analysis into qualitative and mixed methods. Such research has taken a historical-geographical approach to understand the role of political, social, and economic influences (Pulido, 1996). PE is an approach related to EJ, rooted in Marxism and focused on the political-economic processes underlying the production of environments and their associated socioenvironmental inequalities (Heynen, 2014; Watts, 2015). Urban PE (UPE) is considered of particular relevance to EJ, as most EJ research has focused on urban areas (Holifield, 2015). Political ecologists have criticized EJ research for lacking theory and focusing on empiricism and methodology (Swyngedouw and Heynen, 2003). However, the integration of normative theories of social justice, political-economic analyses, and the use of social-movement theory, has aligned EJ closer with PE (Holifield, 2015). Despite emerging in different contexts, both EJ (initially focused on urban America) and PE (rooted in the rural Global South) have expanded to consider a wider range of environmental concerns and an expanding geography (Schlosberg, 2013; Holifield, 2015). Related concepts such as climate, energy, and food justice have emerged and underscore recent EJ focus on materialism and praxis, the role of community and place attachment, and the relationship of humans to non-human elements of nature (Agyeman et al., 2016).

### 3 The Emergence of Green Gentrification Research

Ecological/environmental/green gentrification has been highlighted as the latest front in the EJ movement (Anguelovski, 2016). *Ecological* gentrification was first used to describe “the implementation of an environmental planning agenda related to public green spaces that leads to the displacement or exclusion of the most economically vulnerable human population — homeless people — while espousing an environmental ethic” (Dooling, 2009) and later to highlight how sustainability narratives have been used to facilitate redevelopment (Quastel, 2009). *Environmental* gentrification was first used to describe gentrification following brownfield redevelopment (Sieg et al., 2004; Banzhaf and McCormick, 2006) and was later popularized as “a process […] which builds on the material and discursive successes of the environmental justice movement and appropriates them to serve high-end development” (Checker, 2011). *Green* gentrification was coined to highlight “urban gentrification processes that are facilitated in large part by the creation or restoration of an environmental amenity” (Gould and Lewis, 2012). Although using different terms, these concepts all broadly focus on the impact of greening actions and sustainability narratives on social-ecological urban environments. Interest in green gentrification has proliferated and recently expanded beyond its original North-American context (Ali et al., 2020; Anguelovski et al., 2017; Chen et al., 2021; Kwon et al., 2017). Many studies have researched improvements to the quality/quantity of urban vegetation, such as parks (Anguelovski et al., 2017; Rigolon and Németh, 2020), urban agriculture (Alkon et al., 2019; Marche, 2015), and greenways/trails (Patrick, 2014; Immergluck and Balan, 2018). Other types of greening such as brownfield and waterfront redevelopment, smart growth/eco-density, green-building certification, and health-food stores, have also been implicated (Pearsall, 2018). To complicate matters further, additional related concepts such as climate (Keenan et al., 2018) and resilience gentrification (Gould and Lewis, 2018) describe the impact of climate change—and responses to it—on gentrification. These concepts differ from green gentrification but can have considerable overlap. For clarity and reduced scope, this paper will use “green gentrification” and focus on vegetative forms of greening and their relation to gentrification.

## II Methods

This review followed PRISMA guidelines (Moher et al., 2009; Page et al., 2021) to systematically collect and review relevant literature on vegetative green gentrification and examine how this phenomenon is being studied. A systematic approach was taken to limit bias and ensure quality during literature collection and data analysis/synthesis.

### 1 Review Approach and Data Collection

This review builds on a previous scoping review conducted by Sax et al. (2021, under review), which created a framework for green gentrification. We use the same conceptualization of green gentrification, which is concerned with the impact of improved quality/quantity of vegetative urban greening on gentrification—although some included studies discussed both vegetative and non-vegetative greening. The original scoping review was current to February 2020, and this review updated the collected studies to October 31, 2021 by re-conducting the same literature-search process. Two authors calibrated the inclusion criteria and screening process by reviewing the first 100 indexed references together before splitting the records for title/abstract review. Together, both authors reviewed the complete text of the first 50 papers selected for full-text review before dividing the remaining papers between them. Both authors discussed their inclusion/exclusion decisions throughout the process to ensure consistency. One author calibrated with the third author for the updated literature search. All three authors discussed inclusion/exclusion decisions and approved the final list of additional included literature. Unfortunately, one database used in the scoping review (Urban Studies Abstracts) was inaccessible for this study, but this is unlikely to be an issue given the relatively small number of journals indexed by Urban Studies Abstracts and the degree of overlap (65%) with those indexed by other databases used (Web of Science Core Collection, GEOBASE, and Econlit). Non-overlapping journals were examined, and none were particularly relevant for green-gentrification literature or returned any papers included in the initial scoping review.

**Figure 1.**
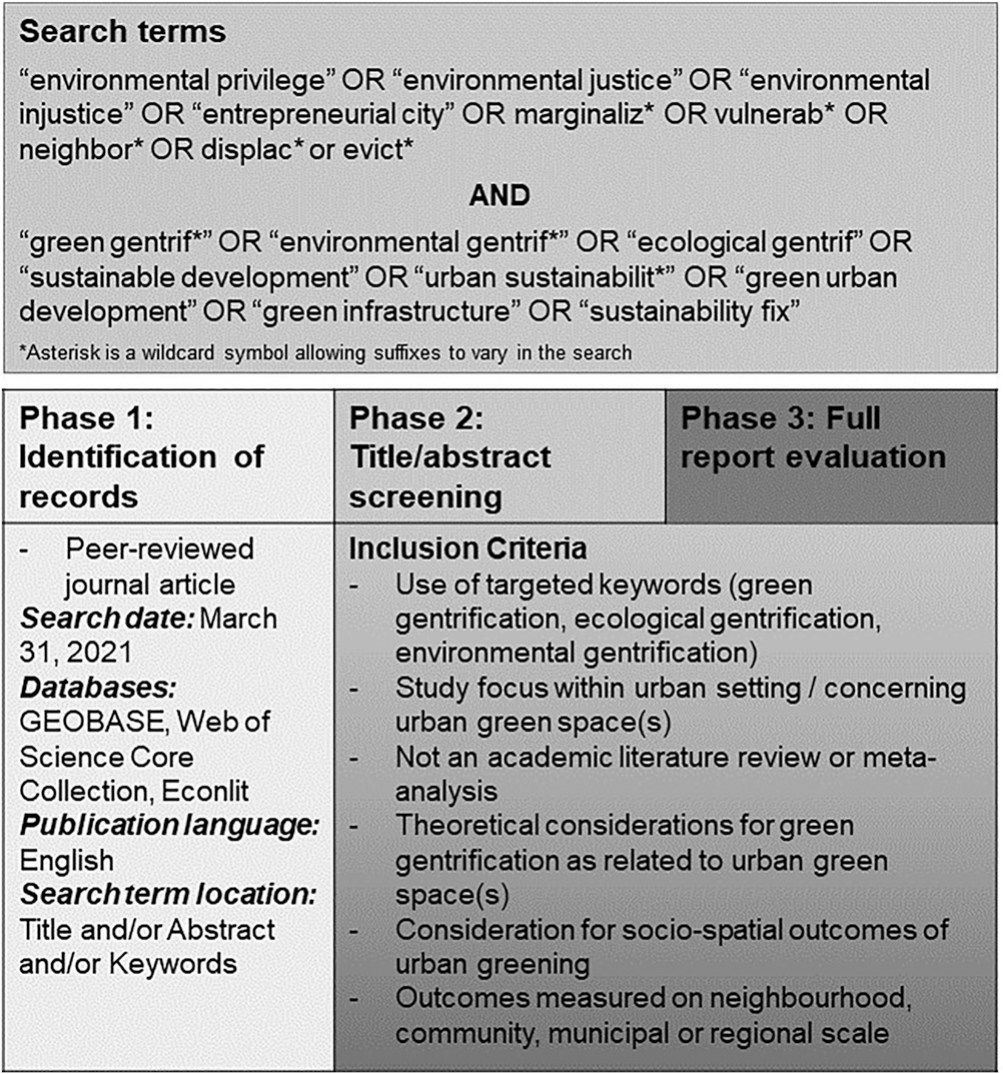
Search terms, inclusion criteria, and literature review process. Adapted from Sax et al. (under review).

**Figure 2.**
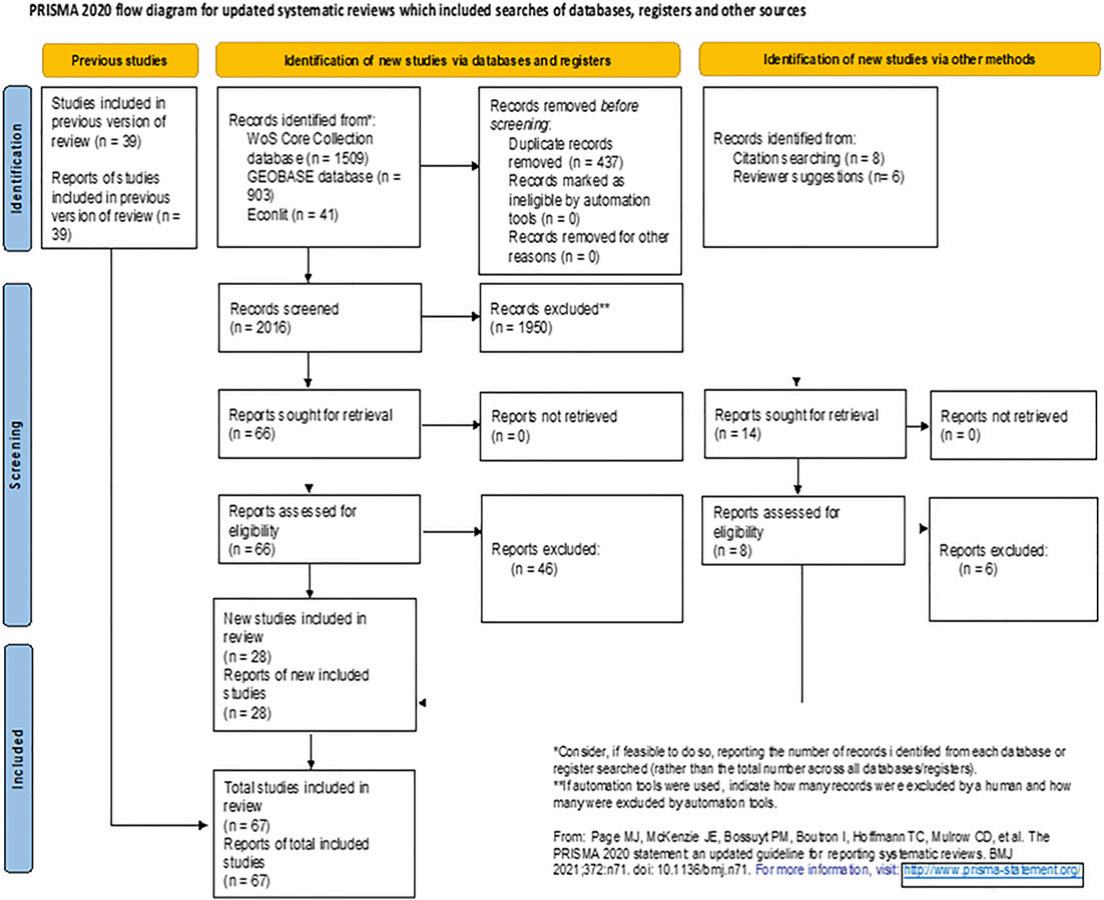
PRISMA flow chart (Page et al., 2021) outlining literature collection and review process.

This review used the same search terms as the scoping review (Figure 1), which were determined based on highly cited green-gentrification literature. It includes synonyms for green gentrification and terms typical of social-justice literature. Similar fields that may also include vegetative greening (e.g., climate and resilience gentrification) were not explicitly included since they are not necessarily interchangeable with environmental/ecological/green gentrification. However, several papers from these fields were returned through our search, and a few were included because of their focus on vegetative greening. An additional eight papers were obtained via snowballing citations from included literature to capture important texts missed by the search terms, for a total of 67 articles (Figure 2). This review did not include all the papers from the scoping review. Since one objective of this study was to determine whether the methods used are sufficient for identifying gentrification and understanding the role of greening, studies that were purely theoretical, academic literature reviews, or meta-analyses were excluded. Furthermore, studies that did not aim to study green gentrification and mentioned it only in the discussion/conclusion were excluded for this same reason.

### 2 Data analysis and Synthesis

All studies were reviewed to collect and record data on the items outlined in Table 1 to facilitate content analysis (Krippendorff, 2004). Categorization of data items took an inductive and iterative approach to creating categories to avoid a preconceived understanding of the methods used to study green gentrification. Some data were only collected for certain studies, such as “Interview subject” data being collected only for studies including interviews.

**Table 1.** Data items recorded during analysis. Any categories used were created based on the data.

Data item	Data recorded
Case study inclusion	Presence/absence
Case study location(s)	City, country
Greening type(s) studied	Multiple categories (general greening, park, greenway, urban agriculture, other)
Data collection/analysis methods	Multiple categories (see Table 2)
Document type	Type of document(s) reviewed
Interview subject	Role/descriptor of interview subjects
Observation focus	Descriptor of observation focus
Spatial-analysis specifics	Temporal span, spatial unit(s), statistical methods
Analytical approach	Qualitative/quantitative/mixed
Theories/frameworks	Any theory/framework used
Gentrification indicators	Any indicator used to identify gentrification (sociodemographic/economic variables, neighborhood change, etc.)

## III Results and Discussion

### 1 Geographic Focus

**Figure 3.**
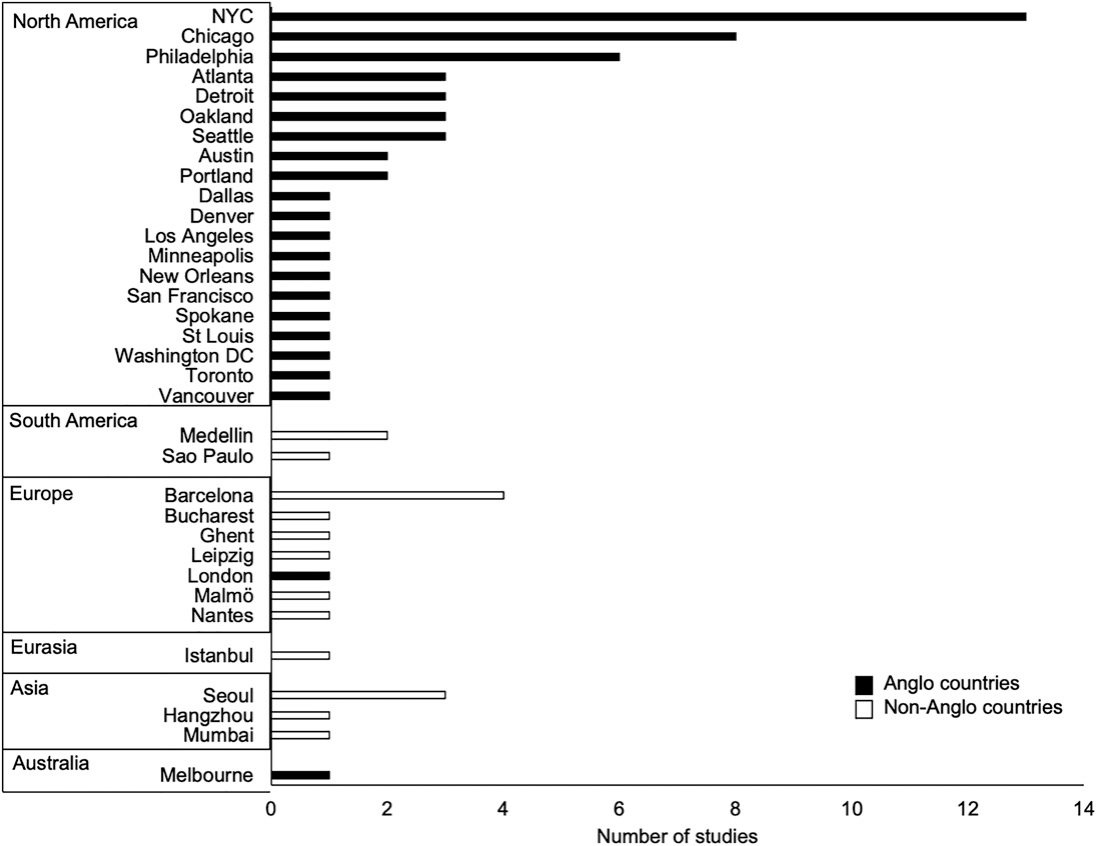
Number of case studies per city, continent, and Anglo context. Total number of cities is greater than total number of included studies as some papers featured >1 case.

Nearly every paper took a case-based approach and focused on green gentrification in 1–3 cities. Most case studies focused on one city (91%) and examined at least one US city (70%) but 16 countries were represented overall (Figure 3). Only 16% of case studies were conducted in non-Anglo contexts, and these were predominately European. The emphasis on US case studies is unsurprising given the plethora of gentrification and EJ research conducted there. Many green-gentrification studies focused on large cities such as NYC, Chicago, Los Angeles, and Philadelphia, which have been the subject of much gentrification research. Some green-gentrification research was also done in historically “shrinking” (some now recovering) cities, including Philadelphia, Detroit, and St Louis (Appendix A; Table A1; see also Ali et al. (2020) for Leipzig, Germany). These latter cases highlight the use of vegetative greening to attract middle-class newcomers and counteract declining populations.

Only one case study (Håkansson, 2018) was based in the United Kingdom, despite the inception and long history of gentrification research in this country (Lees et al., 2008). The lack of research from South America, Asia, and Africa is also apparent but less surprising. There has been much debate in gentrification research about the concept’s applicability to South/East contexts (Bernt, 2016). This was highlighted in the case of Mumbai, where the neoliberal market-based emphasis of green gentrification was deemed insufficient to explain the city’s state-sanctioned eminent-domain redevelopment processes (Doshi, 2019). Even within Western contexts there can be large differences in gentrification: the stereotype of White gentrifiers displacing predominately Black/Latino populations is a US-centric model that is less applicable in, for example, the UK (Lees, 2016). Some non-Anglo countries have their own terms to describe similar processes to gentrification, such as *embourgeoisement*, used to describe urban change in Paris (Préteceille, 2007). This literature review included only English-language articles, and it is likely that non-Anglo research on concepts similar to green gentrification exists under different names.

### 2 Methods in Green-Gentrification Research

Over 55% of studies took a qualitative approach. Over half used interviews (Dooling, 2009; Fernandez et al., 2019; Romero and Harris, 2019), and a third used observations (Montgomery, 2015; Alkon and Cadji, 2020; Harris et al., 2020b). There were relatively few surveys, and these were quantitatively designed (Mullenbach et al., 2019; Oscilowicz et al., 2020). Most studies interviewed multiple stakeholders, and frequent interviewees are outlined in Table 2. There could be overlap between some of these groups (e.g., residents and activists), and this may not have been explicit. Few studies interviewing residents specified whether they were long-term residents or recent newcomers (but see Alkon et al., 2019; Curran and Hamilton, 2012; Goossens et al., 2019; Harris et al., 2020a; Oscilowicz et al., 2020) which has implications for understanding gentrification processes and outcomes. Relatively few interviews were conducted with real-estate developers (but see Safransky, 2014; Garcia-Lamarca et al., 2019; Shokry et al., 2021) despite this being a prominent group involved in the public-private partnerships commonly implicated in green gentrification. Observational studies focused on participants in greening initiative(s) of interest (Loughran, 2014; Oscilowicz et al., 2020), meetings pertaining to them (Dooling, 2009; Checker, 2011; Safransky, 2014), and site characteristics of greening and its surroundings (Håkansson, 2018; Ali et al., 2020; Pearsall and Eller, 2020).

**Table 2.** Methods employed in green-gentrification research. Total number of studies exceeds number included in the review as most papers used multiple methods.

Method	Type/source/focus	Number of studies
Document/other review	Academic literature	5
	Archives	10
	News media	22
	Policy/planning documents	20
	Social media	4
	Technical reports	9
	Websites	10
	Other	15
	Unspecified materials	4
Total		36
Interviews	Activists	11
	Business owners/employees	11
	City officials	19
	Developers	3
	NGOs/community organizations	12
	Residents	18
	Other	10
Total		35
Observations	Greenspace users	11
	Meetings	6
	Site	9
	Other	2
Total		23
Spatiotemporal analysis	Correlation analysis	3
	Difference-in-differences	3
	Geographically weighted regression	2
	Hedonic models	3
	Hotpot analysis	3
	Spatial autoregressive models	3
	Other	8
Total		22
Surveys		2

Qualitative approaches also included review/analysis of various materials, primarily news media (Curran and Hamilton, 2012; Garcia-Lamarca et al., 2019; Rigolon et al., 2020b) and planning/policy documents (Checker, 2011; Safransky, 2014; Glennie, 2020) but also websites and social media (Håkansson, 2018; Amorim Maia et al., 2020; Parish, 2020) and archival materials (Bryson, 2013; Draus et al., 2020; McNeur, 2017). Typically, case studies employed a combination of various review materials, often in addition to interviews and/or observations, resulting in a contextually rich understanding. Researchers have highlighted the importance of context in gentrification (Maloutas, 2012), EJ (Walker, 2009) and PE (Loftus, 2019), indicating the need to recognize the role of geography, politics, economy, culture, and history at multiple spatial and temporal scales. However, there is an implicit trade-off between the number of data sources and methods included and the depth to which they can be analyzed and discussed.

Spatiotemporal analyses were less frequently employed than qualitative approaches but were used in nearly a third of studies. Such analyses were all published between 2017 and 2021 and aimed to understand the scope and extent of green gentrification (as suggested by Anguelovski et al., 2019). There is a long history of spatial analysis in EJ and earlier political-economic studies of greening and brownfield redevelopment (Sieg et al., 2004; Banzhaf and McCormick, 2006). Most studies used socioeconomic/demographic and real-estate indicators to identify gentrification within buffer areas surrounding a greening initiative(s). Multivariate-regression analyses were typical, and census tracts/blocks were the usual spatial unit of analysis. Some studies took a time-series approach and compared how indicators changed over time (Anguelovski et al., 2017; Braswell, 2018; Rigolon and Németh, 2020). Others employed a difference-in-difference approach, where “treatment” and “control” groups (determined based on proximity to greening) were compared to control for site and time-specific factors and make more robust causal inferences (Park and Kim, 2019; Black and Richards, 2020; Sharifi et al., 2021). Changes in indicator variables were typically examined over one time period, usually 5–10 years in length, occurring sometime between 2000 and 2015. The narrow temporal range is likely due to (1) lack of longitudinal census/greening data; (2) discrepancies in census data/geography over time; and (3) widespread interest in urban greening (and green gentrification) being more recent. However, a qualitative study of antebellum Manhattan highlighted how parks have historically commanded higher real-estate premiums and been used to “clean up” disinvested areas (McNeur, 2017), suggesting this last point does not confirm green gentrification to be only a recent phenomenon.

Quantitative gentrification studies have typically used indicators based on ethnicity, education, income, professional status, and housing costs to identify the inmoving of professionally employed, high-income, White individuals (Freeman, 2005; Ding et al., 2016). As mentioned above, much gentrification research is rooted in the US, resulting in the repeated use of certain variables. Researchers outside the US need to carefully consider which variables best represent gentrification in their cities, such as single elderly residents in Barcelona (Anguelovski et al., 2017), or non-agricultural workers in Hangzhou (Chen et al., 2021) to recognize those vulnerable to displacement. There could also be variation within individual countries, as for example, economic and ethnic profiles in Canada vary greatly amongst cities. What has yet to be seen in spatial analyses of green gentrification is an operationalization of displacement—a notoriously difficult phenomenon to quantify (Easton et al., 2020). Not only is there a lack of available data on the movement of individuals, displacement can take psychological forms that do not necessarily result in physical dislocation (Zhang and He, 2016). It is also difficult to determine whether people have moved voluntarily or involuntarily and whether involuntary moves are the result of gentrification.

### 3 What is Green Anyway?

It was often unclear whether green gentrification was a result of greening characteristics themselves, the spectacle of greening, the initial (and planned future) characteristics of the surrounding neighborhood, or something else. Many studies discussed urban vegetation in general without focusing on specific examples/types (33%) while others studied greenways (31%), other parks (21%), urban agriculture (11%) or “other” greening, such as living streets (Goossens et al., 2019) and nature preserves (Sandberg, 2014) (Appendix A). Despite extensive academic literature on urban forests, only two studies (Parish, 2020; Donovan et al., 2021) emphasized trees. The emphasis on greenways is particularly notable. Earlier green-gentrification research included many case studies of the NYC High Line, which may have inspired later emphasis on greenways in cities such as Seoul and Chicago. Early emphasis on the High Line is understandable, as the project underscores many recurring themes in green-gentrification research: neoliberalism, real-estate speculation, public-private partnerships, tourist appeal, racialized spatial planning, and brownfield redevelopment. Further, a study of 10 American cities found that greenways (vs. other parks) are more associated with gentrification (Rigolon and Németh, 2020). The urban-agriculture literature is an interesting contrast, as it indicates how smaller, community-based initiatives can be co-opted to create landscapes for privileged consumers to the detriment of local residents (Alkon et al., 2019; Braswell, 2018; Glennie, 2020; Maantay and Maroko, 2018). This is in some ways reminiscent of sweat equity versus state-led and new-build gentrification (Lees, 2012) and highlights that green gentrification can take diverse forms.

Few studies directly compared how the function and/or quality of different types of greening may influence gentrification (but see recent work by Amorim Maia et al., 2020; Chen et al., 2021; Kim and Wu, 2021; Pearsall and Eller, 2020; Rigolon and Németh, 2020) although many earlier studies made inferences about it (e.g., Anguelovski et al., 2017; Johnson Gaither, 2019). Although this study focused on vegetative greening, some papers also discussed other types of greening alongside vegetation, such as LEED-certified buildings (Checker, 2011; Yazar et al., 2020), cycle lanes (Goodling et al., 2015; Ali et al., 2020), and public transit (Quastel, 2009; Rigolon et al., 2020b), highlighting the link between vegetative and non-vegetative drivers of green gentrification. It is easy to see potential overlap between green gentrification and climate and resilience gentrification. However, overlap with other types of gentrification such as transit (Padeiro et al., 2019), rural (Phillips, 1993), commercial (Zukin and Kosta, 2004), tourism (Gotham, 2013), and super gentrification (Lees, 2003) is also probable. Some researchers noted such links (Loughran, 2014; Park and Kim, 2019; Alkon and Cadji, 2020; Oscilowicz et al., 2020), but further exploration/discussion are warranted regarding how related types of gentrification can be studied more holistically.

### 4 Theories and Frameworks

Multiple theories and frameworks have been adapted from related fields and applied to green gentrification. The urban growth machine (Molotch, 1976) has been re-envisioned as a “green growth machine” promoting green economic growth that will purportedly benefit everyone through trickle-down effects (Glennie, 2020; Loughran, 2014; Lang and Rothenberg, 2017; Mullenbach et al., 2021; Rigolon and Németh, 2018). The “sustainability fix” (While et al., 2004) describing how environmental objectives are selectively integrated into urban planning as a means of encouraging capital accumulation (Curran and Hamilton, 2012; Goodling et al., 2015; Montgomery, 2015). Rent-gap theory (Smith, 1979, 1987) has been rebranded as a green/environmental/ecological rent gap to explain how governments/developers use greening to close discrepancies between realized and potential ground rent (Anguelovski et al., 2018; Braswell, 2018; Quastel, 2009; Yazar et al., 2020). Creative-class theory (Florida, 2002) has highlighted how cities use greening to attract upwardly mobile creative/technological workers (Safransky, 2014; Marche, 2015; Montgomery, 2015). Racial capitalism (McClintock, 2018) has elucidated the role of historical-geographical racialization in the valorization of urban greening (Glennie, 2020; Shokry et al., 2021). Some unique theoretical applications have been queer theory (Patrick, 2014), Just City Theory (Connolly, 2019), settler-colonial theory (Safransky, 2014; Parish, 2020), Debord’s theories on spectacle (Sandberg, 2014; Langhorst, 2015), and stigma theory (Harris et al., 2020b). This wide range of theories indicates the many ways of understanding and interpreting green gentrification and highlights a diversity of potential mechanisms. Such diversity and pluralism have been noted in gentrification research, for example, in the edited volume titled “Global gentrification **s**” (Lees et al., 2015). Given the multiplicity of greening types and drivers implicated so far, it is useful to consider green gentrifications instead of a singular green gentrification. Kocisky (2021) has begun such work by highlighting the plurality of (in)justices surrounding green gentrification. Overall, it was not always clear what theoretical assumptions or frameworks were applied in green-gentrification studies. Many papers presented an introductory overview including reference to some of the aforementioned theories but did not explicitly state whether they were applied in their own research. Increased epistemological transparency within this emerging field will allow targeted critique, challenges to existing theoretical assumptions, and more fruitful discussion overall amongst scholars.

### 5 Are we Really Studying “Green Gentrification”?

**Figure 4.**
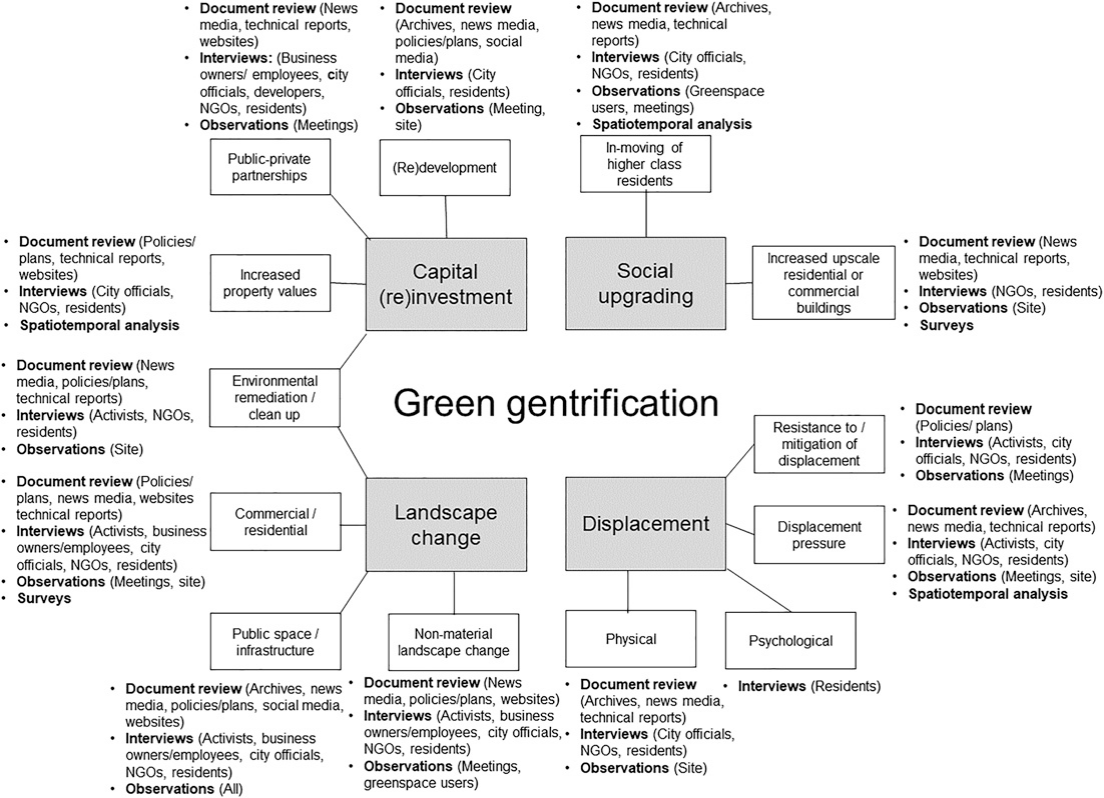
Methods used to capture the four characteristics of green gentrification.

Gentrification is a highly political term, and its application to greening, which is often perceived or promoted as apolitical, can garner attention to social injustices influenced by these acts. However, the debates surrounding what constitutes gentrification are a reminder that it cannot be attached to every study of increased real-estate value surrounding greening initiatives. Although green-gentrification research should avoid becoming too bogged down in semantics, there needs to be some conceptual clarity on what constitutes gentrification and how greening is implicated in its processes and outcomes. Gentrification is complex to define, identify, and understand—in part because of the evolution and multiple mutations of gentrification. Four broad characteristics of contemporary gentrification were identified to assess new-build gentrification as one such mutation: (1) capital reinvestment; (2) social upgrading^2^ of an area by high-income in-movers; (3) landscape change; and (4) displacement of low-income groups (Davidson and Lees, 2005). This list may not be definitive and should evolve over time, but it provides a definition that is “targeted but elastic” (Clark, 2004; Lees et al., 2008). It is used here as a framework to consider the evidence for green gentrification in the literature and how this evidence was captured (Figure 4).

#### 5.1 Capital Reinvestment

Capital reinvestment has been implicated in green gentrification primarily through discussions of real-estate speculation, green rent gaps (Braswell, 2018; Quastel, 2009) and public-private partnerships (Anguelovski et al., 2017; Safransky, 2014). It is often operationalized as a gentrification indicator via increased real-estate values and the extent of surrounding redevelopment. It is suggested that greening serves as an anchor for capital reinvestment and redevelopment (Anguelovski et al., 2018), but greening can also be an indicator of capital reinvestment. Many studies implicated greening in attracting capital reinvestment, but it is less clear whether greening is the starting point for such reinvestment or how much reinvestment would occur without it. Some studies have noted gentrification already occurring nearby prior to greening (Loughran, 2014; Pearsall and Eller, 2020) and more park funding directed to areas already experiencing gentrification (Reibel et al., 2021). Even if greening is not the starting point for capital reinvestment, it is plausible for it to be situated somewhere within the complex web of capital flow, spurring subsequent reinvestment. However, it is necessary to consider other redevelopment plans and how they influence capital reinvestment (Checker, 2011; Lang and Rothenberg, 2017; Ali et al., 2020; Draus et al., 2020). If a city plan includes greening alongside several other developments, it becomes less clear how much influence greening has played in attracting capital reinvestment. The relative importance of greening as a factor driving gentrification likely differs depending on the case, adding difficulty in understanding its role more broadly. Several researchers implicated public-private partnerships in green gentrification based on interviews, observations, document review, etc., but it is not always clear how such partnerships mobilize capital reinvestment in their specific context. Those who elucidated such partnerships described local governments enabling zoning changes for redevelopment to boost city tax revenue (Checker, 2011; Millington, 2015) and the creation of private/semi-private parks (Lang and Rothenberg, 2017; Pearsall and Eller, 2020). However, public-private partnership could include a range of actors and processes, which have not been fully explored.

#### 5.2 Social Upgrading

Social upgrading has been the most frequently studied characteristic and informs many indicators used to identify gentrification surrounding greening (race/ethnicity, household income, education, real-estate values, etc.). Most spatiotemporal analyses were designed to show how sociodemographic/economic factors changed surrounding greening initiatives by comparing these indicators before and after greening (Anguelovski et al., 2017; Immergluck and Balan, 2018). The indicators selected (and in quantitative studies, their thresholds) determine which areas are identified as gentrifying. As discussed previously, indicators have to be contextually and theoretically relevant such that they represent change within a particular context. Beyond standard indicators, some have justified the inclusion of age (Romero and Harris, 2019), gender (Pearsall and Eller, 2020), and country of origin (Anguelovski et al., 2017). As in earlier quantitative gentrification studies (Ding et al., 2016; Freeman, 2005), some green-gentrification studies used criteria to determine a priori which spatial units are “gentrifiable” (Anguelovski et al., 2017; Rigolon and Németh, 2020; Shokry et al., 2020). Some studies used only income for this a priori designation, which could miss other factors that make an area susceptible to gentrification—such as building stock, historical status, proximity to other gentrifying areas/CBD. More broadly, this approach is guided by the notion that not all areas within a city can gentrify, as some already comprise the demographics commonly associated with gentrification (e.g., wealthier individuals). The a priori designation of gentrifiable tracts, as well as identification of gentrification based on thresholds of change in indicators, suggest identifiable start and end points for gentrification—in contrast to the notion that gentrification is an ongoing process differing across time and space (Lees et al., 2008). Deciding which areas can gentrify also ignores phenomena such as “super-gentrification” (Lees, 2003), and while some might dismiss the notion of gentrifiers being replaced by wealthier gentrifiers, and maybe even view it as comeuppance, the potential for trickle-down or knock-on gentrification in other areas is concerning. State-led gentrification has resulted in gentrification of areas previously thought un-gentrifiable (Mösgen et al., 2019), further suggesting a priori identification of gentrifiable areas paints an incomplete picture. Several qualitative studies used census data or interviews and observations to describe social upgrading (Alkon and Cadji, 2020; Checker, 2011; Goodling et al., 2015; Loughran, 2014), although Romero and Harris (2019) highlight differences between how long-term residents perceive the class of newcomers versus how these newcomers perceive their own class position. While many studies used social upgrading to inform indicators, others also discussed how social upgrading influences further neighborhood change through shifts in power dynamics, prevailing desires, displacement pressures and more (Alkon et al., 2019; Goossens et al., 2019; Harris et al., 2020a). This is an important contribution and acknowledges that social upgrading is not only an outcome of gentrification but can also influence further gentrification.

#### 5.3 Landscape Change

Landscape change in gentrification has often focused on material landscapes (Phillips, 2015). Green gentrification has highlighted how landscapes can encourage or discourage gentrification, and how the landscape of the post-industrial city has changed to meet current demand for greener urban environments. Material landscape change occurs from the creation/upgrading of greening itself as well as surrounding redevelopment. As discussed previously, multiple greening types have been studied, but there has been little examination of how greening type influences gentrification and further landscape change. When considering landscape change, many studies acknowledged other nearby (re)developments (cycle lanes, LEED-certified buildings, sustainable transport, cafés, etc.), but there has been little discussion about the interplay between these amenities and greening (but see Ali et al., 2020; Bryson, 2013; Checker, 2011). It is typically unclear whether these amenities have been established before, alongside, or following greening although it is often implied to be following. Similar to capital reinvestment, it is difficult to place greening within the process of landscape change because greening can be both influenced by previous landscape change and influence future landscape change. Greening can be very visible and highly publicized, which may obscure consideration of other landscape change. This is further complicated by potential discrepancies in timelines of conception versus implementation. If greening was conceived alongside other developments but implemented before them, this may give the impression that greening spurred subsequent landscape change. This is difficult to tease out in green-gentrification research, as the conception timeline of other developments may not be public knowledge. Some (typically qualitative) studies used landscape change as a gentrification indicator by referencing the arrival of new high-end residential developments, restaurants, cafés, and boutiques (Sandberg, 2014; Lang and Rothenberg, 2017; Parish, 2020). Using landscape change as an indicator requires the same contextual and theoretical considerations as those based on social upgrading, and it has to (1) reflect change that accommodates gentrifiers instead of long-term residents and (2) acknowledge that gentrifier preference may vary across contexts. Beyond material landscapes, some green-gentrification research considered how landscapes reflect social life and power relations, indicating that landscape change is not just a material manifestation of capital (re)investment. Several studies used interviews, observations, and document analysis to identify and discuss the actors involved in landscape change, including gentrifiers (Curran and Hamilton, 2012; Goossens et al., 2019), business owners (Romero and Harris, 2019; Parish, 2020), developers (Quastel, 2009; Safransky, 2014), grassroots organizations (Håkansson, 2018), and governments (Anguelovski et al., 2017; Doshi, 2019). It is important to understand not only who is involved but the means by which they participate in and inform landscape change—a point some highlighted as an issue of procedural justice (Mullenbach et al., 2019; Rigolon et al., 2020a). Some discussed landscape change as symbolic and reflective of cultural values of sustainability (Patrick, 2014; Langhorst, 2015; Lang and Rothenberg, 2017; Garcia-Lamarca et al., 2019). Others highlighted that how landscapes are lived reflects for whom they are intended (Checker, 2011; Loughran, 2014; Safransky, 2014; Harris et al., 2020a). Attention to non-material (social, symbolic, lived, representational, etc.) landscapes in green gentrification was often not specified as a research objective but emerged in explanations of how and why the material landscape changed.

#### 5.4 Displacement

Displacement has been the least-studied characteristic despite being the main rationale for why gentrification constitutes an injustice. Displacement in green gentrification is typically conceptualized as physical or psychological displacement of marginalized residents due to gentrification processes following greening—not the greening itself. While most researchers mentioned the potential for displacement in green gentrification, its actual occurrence was infrequently captured except in obvious cases such as residential demolitions (Bryson, 2013; Dooling, 2009; Doshi, 2019) and through second-hand information in interviews (Oscilowicz et al., 2020). Typically, displacement (or the threat of future displacement) in green gentrification has been inferred based on indicators of social upgrading (Immergluck and Balan, 2018; Maantay and Maroko, 2018) and increased housing/living costs (Fernandez et al., 2019; Alkon and Cadji, 2020). The lack of evidence could also be due to the relatively recent development of some greening initiatives studied, as insufficient time may have passed for displacement to be evident. Although much focus has been on physical displacement due to increased costs, some used interviews and observations to highlight psychological displacement and exclusion (Millington, 2015; Harris et al., 2020a; Rigolon et al., 2020a). Several texts have identified and described typologies of displacement seen in gentrification (Elliott-Cooper et al., 2020; Zhang and He, 2016), many of which have not been explicitly discussed within green gentrification (but see Ali et al., 2020; Goossens et al., 2019). However, there are efforts outside of academic literature that aim to capture displacement from green gentrification (see the Urban Displacement Project, for example). Similar to the other gentrification characteristics, displacement could be viewed as both a cause and outcome. Much focus has been on displacement as an outcome, but the displacement of “undesirable” neighbors could attract further inmoving of gentrifiers. 

#### 5.5 Consideration of all four characteristics

None of these four characteristics can be considered the sole cause, outcome, or indicator of gentrification, and all can occur in its absence. Green-gentrification research needs to concern itself with how greening influences, and is influenced by, each of them. While the early, highly cited studies of environmental/ecological/green gentrification (Dooling, 2009; Checker, 2011; Gould and Lewis, 2012) captured elements of each of these four characteristics, the definitions tend to emphasize only one or two. It may be useful to conceive of green gentrification as a process in which capital (re)investment and greening create landscape change geared towards a higher class of residents, resulting in displacement of marginalized households. There is a lack of exploration of the mechanisms through which greening facilitates gentrification, which are necessary for further refining the definition of green gentrification. Many discussed green rent gaps and the use of greening to legitimize redevelopment and continued economic growth, but greenwashing is unlikely to be the sole mechanism. Less attention has been paid to consumer preference and whether gentrifiers move to an area because of greening or other redevelopments implemented alongside it. If the move is due to greening, is it because of its form/function, the aesthetic or spectacle, the pretense of sustainability, or something else entirely? Understanding such rationales will inform more nuanced solutions for limiting gentrification in the future.

## IV Future Directions

### 1 Expanding Case Studies

Most green-gentrification research used case studies of American cities, rooting our current understanding in an American context. Expanding green-gentrification theory necessitates a wider geographical context—with some careful consideration. There has been debate about applying gentrification to non-Anglo/Western contexts, with concerns about colonialism, misclassification/obscuring other phenomena, and whether gentrification is truly planetary (Bernt, 2016). Researchers need to avoid superimposing an American conception of green gentrification onto other contexts, as some processes and outcomes will differ. There has been interest in utilizing comparative urbanism in gentrification studies to systematically identify similarities and differences between cities to understand that which exists between what is true for all cities and what is true for one city at one time (Nijman, 2007). Little guidance exists for how to achieve such comparison, and some attempts have still assumed gentrification migrated from North/West to South/East (Lees, 2012, 2018). Green-gentrification research should look beyond cities frequently featured in gentrification discussions, shed the hierarchical classification of cities and assumption of a migrating gentrification template (Lees, 2012, 2018), and pay attention not only to outcomes but also underlying processes and mechanisms (Maloutas, 2012). Similar to theorizing about EJ (Schlosberg, 2004), our understanding of green gentrification can aim to be theoretically broad, locally grounded, and plural. A plural understanding recognizes that processes and outcomes differ depending on context, which will help identify more appropriate and nuanced solutions.

Future case studies should include greening initiatives with differing characteristics/functions to determine how they influence gentrification. For example, one tenet of the “Just Green Enough” hypothesis is that smaller parks could limit gentrification but current empirical evidence shows mixed support (Rigolon and Németh, 2020; Chen et al., 2021; Kim and Wu, 2021). Greenways have been the most-studied greening type, with much focus on the NYC High Line and similar high-profile projects in other large cities. While these may represent the pinnacle of green gentrification, they tell us about processes and outcomes of a specific greening type (which tend to receive much spectacle and attention). Are parks with no active-transportation function associated with gentrification to the same extent? If they are both associated with gentrification, does it look different in terms of who is gentrifying and how the landscape changes? Does gentrification occur around greening that receives less media attention? Finally, some vegetative greening types have been understudied. Street trees (see Donovan et al., 2021) and green roofs, for example, could signal capital (re)investment despite not having the same use value as a park, greenway, or community garden.

Most case studies focused on occurrences of gentrification surrounding greening, but we can also learn from cases in which gentrification does not occur. Studies comparing gentrifying and non-gentrifying outcomes have highlighted the role of social-cultural associations with greening (Amorim Maia et al., 2020), location and surrounding park context (Rigolon and Németh, 2020) and local government intervention and redistribution policies (Garcia-Lamarca et al., 2019). Such research informs how greening can be implemented to limit gentrification. Case selection should also continue to seek examples of resistance by local residents and non-governmental entities to highlight how gentrification can be limited without government intervention or how residents can successfully lobby government to take appropriate measures (Alkon et al., 2019; Curran and Hamilton, 2012).

### 2 Spatial and Temporal Considerations

Green gentrification, much like broader gentrification research, has focused on outcomes at the neighborhood level. The consideration of processes and influences are typically at the city scale—other than the consideration of neoliberalism on a global scale. There is less consideration of regional, federal, or other scales, which could be due to the high autonomy of American cities. However, there are state and federal policies/institutions that still have influence (see Kern and Kovesi (2018) for the role of federal anti-immigration rhetoric and policy). Individual/household health data (see Cole et al., 2019) opens up avenues for studying displacement and identifying within coarser scales (e.g., census tracts) who is actually vulnerable. Additional consideration of different greening sites within cities/neighbourhoods (Amorim Maia et al., 2020; Pearsall and Eller, 2020) will highlight greening characteristics/functions most likely to influence gentrification. Considering scales beyond global and local becomes increasingly important as the geographical context of research expands, as such spatial scales may have more (or at least different) influence than in the US. The consideration of spatiality is not only about what is happening at different scales but how different scales and places interact with and relate to each other—Anguelovski et al. (2019) also highlighted the need to consider financial/policy flows under a planetary green-gentrification lens. For example, the success of the NYC High Line prompted the creation of similar infrastructure reuse parks in other American cities (The High Line Network, 2021). While there are power dynamics influencing the directionality of policy flow between spatial scales and places (Lees, 2012), it should not always be assumed to be unidirectional within green gentrification. It is also important to consider the form(s) and extent to which global neoliberalism manifests at lower spatial levels to influence local gentrification outcomes and processes (Brenner et al., 2010).

The temporal extent of green-gentrification research has been limited and largely focuses on the 21^st^ century (particularly in quantitative spatiotemporal analyses). Data limitations make longer temporal breadths difficult to achieve, but some have used archival resources to highlight processes akin to green gentrification in the 19^th^ and 20^th^ centuries (Bryson, 2013; McNeur, 2017). Others highlighted the role of historic urban planning/policies—such as redlining—in shaping green gentrification (Draus et al., 2020). As Anguelovski et al. (2019) highlighted in their review, historicity is critical for understanding how greening came to influence gentrification. The temporal spans considered influence conclusions drawn about processes, mechanisms, and outcomes: short time spans may be insufficient to see displacement following greening or to identify landscape changes or other events occurring prior to greening that also influence gentrification. For example, one study found park funding was more likely to be directed towards areas already experiencing gentrification (Reibel et al., 2021), complicating the notion of greening anchoring gentrification. Green gentrification will continue to evolve over time—not only can green space change, so can the meaning and value we ascribe to greening (Angelo, 2021). Beyond greening, city landscapes are altered, planning/policy approaches fall out of favor, economic conditions change, and so on. As these interconnected systems and influences evolve, they alter each other and will ultimately influence gentrification differently. Finally, it is important to acknowledge that temporality and spatiality are interconnected: all case studies of green gentrification describe the phenomenon in a particular space and time. It is not always clear how gentrification unfolds, migrates, and evolves across space and time; thus it is difficult to use our current knowledge of green gentrification to predict what may happen in other cities in the future.

### 3 Learning from Experience: New, Long-Term, and Displaced Residents

There has been relatively little engagement with those experiencing green gentrification, and increased use of interviews/surveys could provide valuable insight into displacement and resident preferences/motivations. Interviews/surveys with long-term residents could provide insight into physical displacement of their neighbors (Oscilowicz et al., 2020) and non-physical displacement/exclusion they themselves are experiencing (Kern and Kovesi, 2018; Harris et al., 2020a). Engagement with physically displaced residents is more difficult and would require strategic recruitment but could potentially be achieved via snowball sampling of current residents and local community groups, as well as disseminating surveys and recruiting for interviews online. There is a need to understand if and how green gentrification is leading to displacement if we want viable solutions to minimize such outcomes. Many have suggested rent control and social housing to minimize displacement, but there needs to be documentation of displacement to motivate political will to implement such measures. Interviews/surveys with new residents could provide insight into their motivations for moving to the area (Goossens et al., 2019). It seems to be largely assumed that gentrifiers move in because of greening, but this has rarely been directly confirmed, and there could be a wide variety of factors motivating individuals to move. This is critical for understanding mechanisms through which greening influences gentrification, as the demand side of green gentrification has not been as well-researched as supply-side arguments about green rent gaps, public-private partnerships, etc. Much green-gentrification research portrays gentrifiers as wealthier, White professionals, which is likely in part due to the importance of racism in shaping American cities. There has been less study of other factors such as gender, sexuality, immigration status, age, family size, green space preferences, and their intersections. Such information may be usefully gleaned from resident interviews. Social media could provide insight similar to participant observation and be useful for understanding resident perspectives, motivations, and resistance (Amorim Maia et al., 2020).

### 4 Selecting Gentrification Indicators

Gentrification indicators need to reflect social vulnerability in a given context and should be informed by previous theory, research, and personal experience, where possible. Using multiple indicators of social vulnerability (vs. relying only on income or real-estate values) provides stronger evidence of displacement pressure. Studies using real-estate values alone tell us only about the effect of vegetative greening on property values—this is of particular relevance as spatiotemporal analyses continue to increase in abundance and the term “green gentrification” gains more traction. Beyond geographical context, indicators need to reflect the plurality, spatiality, and temporality of gentrification. For example, Starbucks has been usefully employed as a gentrification indicator (Hwang and Sampson, 2014) but so have trendy independent cafés (Alkon and Cadji, 2020). This may indicate differences in gentrifiers and their preferences across time and space. There also needs to be reflexivity in the selection of gentrification indicators. Although there are mainstays, such as household income, as gentrification evolves, indicators may need to change to reflect new processes/outcomes. For example, if tourism is a relevant gentrifying force in the area surrounding greening (Lang and Rothenberg, 2017; Oscilowicz et al., 2020), then an increase in short-term rentals and other tourist infrastructure may become relevant indicators. Whether researchers use the four characteristics of gentrification mentioned above as their starting point, having multiple gentrification indicators provides more nuanced insight. This is particularly true when the indicators highlight social, cultural, and physical changes, as gentrification is not merely the in-moving of wealthier residents but all the associated changes alongside it.

### 5 Gentrification Beyond Vegetative Greening

Jane Jacob’s critique of orthodox urban planning underscored that the value and use of parks depends not only on their characteristics but also on their surroundings, including the wider greening context and other aspects of the built environment (Jacobs, 1961). It stands to reason that creating/upgrading a green amenity in a park-rich area will have less gentrifying influence than in a park-poor area, however, this has rarely been explicitly studied (but see Rigolon and Németh, 2020). Beyond surrounding greening context, there needs to be consideration of other factors involved in gentrification in the surrounding area. Gentrification mutations highlight the diverse drivers and agents of gentrification (tourism, public transit, post-secondary institutions, upscale retail, condos, etc.). It seems probable that vegetative greening is not always—if ever—acting alone in promoting gentrification, particularly now that state-led gentrification of large areas has become the norm (Lees, 2016). This is likely particularly true in cities embracing densification and smart-growth policies (which can also be touted as greening—albeit a non-vegetative form) that emphasize mixed-use zoning and place many amenities alongside each other. Not every driver will have the same level of influence, but it is worth considering what other factors are contributing to gentrification surrounding greening. The use of city planning documents, zoning maps, property sales deeds, city permits, media articles, etc., could be useful here. Perhaps a larger question is whether “green gentrification” is a separate entity or if this is simply what gentrification looks like in an era of sustainability narratives and green capitalism. Is gentrification still occurring *without* some form of greening? Many cities have requirements for (re)developments regarding energy efficiency, green space provisions, etc., which may be giving a green face to gentrification.

## V Conclusions

Green-gentrification research thus far has drawn on case studies predominately located in American cities. Early research emphasized qualitative approaches using interviews, observations, and document review/analysis, but recent research has seen an increase in quantitative spatiotemporal analysis assessing the scope and extent of the phenomenon. Advancing green-gentrification theory requires expanding to new geographical contexts without superimposing our current American-based understanding. Further research on the role of greening characteristics/function, as well as studying cases in which gentrification does not occur surrounding greening, will provide insight into how to green cities while limiting gentrification. Considerations of spatiality and temporality are necessary, as methodological choices about space and time have direct impacts on our understanding of green gentrification. To complement existing case work, observations, and document review, more engagement with residents through interviews, surveys, and social media will provide insight into displacement, gentrifier preference/motivation, and potentially highlight some overlooked characteristics of the gentrified and gentrifying. Documenting displacement is critical for motivating implementation of viable solutions, and increased understanding of gentrifier preferences is necessary for understanding the demand-side argument of green gentrification. Selection of gentrification indicators is incredibly important when studying green gentrification, as these are the basis for determining whether gentrification has occurred. Current research relies heavily on indicators of social upgrading (such as household income, education, etc.), and there has been very little evidence of displacement despite this being the major concern of gentrification. With the term “green gentrification” being used in more spatiotemporal analyses, there is need to avoid misrepresenting studies showing only an increase in property values surrounding greening. Finally, if we wish to obtain a nuanced understanding of green gentrification, we need to consider other potential factors influencing gentrification alongside greening and whether green gentrification is a separate entity or if gentrification is always “green” these days.

Many cities are promoting sustainability agendas that prioritize greening. While urban greening provides myriad benefits, the potential for gentrification and displacement threatens to undermine even well-intentioned efforts to green previously underserved communities. At the same time, cities are growing and changing systems that need to adapt to multiple and intertwined challenges, such as climate change, inequality, and (un)sustainability; communities need to undertake greening that supports urban well-being and justice. This necessitates an understanding of what is driving green gentrification, how it occurs, its outcomes, and ways in which it has been successfully limited in practice. Achieving this improved understanding requires researchers to expand the methods and methodological considerations applied to our research. With this being such a rapidly emerging field, there is no doubt that at least some issues and potential avenues for future research suggested here are currently being addressed in ongoing research, and the authors look forward to the dissemination of such pursuits.

## Notes

1 It is important to note displacement can take many forms, both physical and non-physical (see (Zhang and He, 2016)).

2 Social upgrading here refers to increases in household income, professional occupation status, and educational attainment.

## Appendix A: Literature included in the systematic review.

**Table A1:** Included papers sorted by greening type and case-study city. “General” indicates study did not focus on a specific type of greening. “N/A” indicates the paper did not include a case-study element. N does not always sum to “Total” as some papers included cases in more than one city.

Greening type	City	N	Citations
General	Atlanta	1	Johnson Gaither (2019)
	Austin	1	Garcia-Lamarca et al. (2019)
	Barcelona	1	Anguelovski et al. (2018)
	Bucharest	1	Rufat and Marcinczak (2020)
	Chicago	3	Kern & Kovesi (2018); McKendry and Janos (2014); Rigolon and Ne meth, (2020)
	Detroit	2	Montgomery (2015); Safransky (2014)
	Istanbul	1	Yazar et al. (2020)
	London	1	Håkansson (2018)
	Medellín	1	Anguelovski et al. (2018)
	Melbourne	1	Sharifi et al. (2021)
	Mumbai	1	Doshi (2019)
	Nantes	1	Garcia-Lamarca et al. (2019)
	New Orleans	1	Anguelovski et al. (2018)
	NYC	4	Checker (2011); Connolly (2019); Curran and Hamilton (2012); Kim and Wu (2021)
	Philadelphia	2	Pearsall and Eller (2020); Shokry et al. (2020); Shokry et al. (2021)
	Portland	1	Goodling et al. (2015)
	Seattle	2	Dooling (2009); McKendry and Janos (2014)
	Vancouver	1	Quastel (2009)
	Total	23	
Greenways	Atlanta	1	Immergluck and Balan (2018)
	Chicago	4	Fernandez et al. (2019) ^a^; Harris et al. (2020a,b); Rigolon & Németh (2018)
	Detroit	1	Draus et al. (2020)
	Leipzig	1	Ali et al. (2020) ^a^
	NYC	7	Black and Richards (2020); Lang and Rothenberg (2017); Langhorst (2015); Loughran (2014); Millington (2015); Mullenbach et al. (2021) ^a^; Patrick (2014)
	Oakland	1	Patterson and Harley (2019)
	Philadelphia	1	Mullenbach et al. (2021) ^a^
	Seoul	3	Kwon et al. (2017); Park & Kim (2019); Schuetze and Chelleri (2016)
	Total	19	
Parks	Atlanta	1	Rigolon and Ne meth, (2020) ^a^
	Barcelona	3	Amorim Maia et al. (2020); Anguelovski et al. (2017); Oscilowicz et al. (2020)
	Chicago	2	Fernandez et al. (2019) ^a^; Rigolon and Ne meth, (2020)
	Dallas	1	Mullenbach et al. (2021) ^a^
	Hangzhou	1	Chen et al. (2021)
	Leipzig	1	Ali et al. (2020) ^a^
	NYC	1	McNeur (2017)
	Philadelphia	2	Mullenbach et al. (2019); Rigolon and Ne meth, (2020)
	São Paulo	1	Baumgartner (2021)
	Toronto	1	Parish (2020)
	N/A	1	Rigolon and Németh (2020)
	Total	14	
Urban agriculture	Austin	1	Romero and Harris (2019)
	Denver	1	Sbicca (2019)
	NYC	1	Maantay and Maroko (2018)
	Oakland	2	Alkon and Cadji (2020); Alkon et al. (2019)
	San Francisco	1	Marche (2015)
	Seattle	1	Glennie (2020)
	St Louis	1	Braswell (2018)
	Total	8	
Other	Ghent	1	Goossens et al. (2019)
	Malmö	1	Sandberg (2014)
	Minneapolis	1	Walker (2021)
	Portland	1	Donovan et al. (2021)
	Spokane	1	Bryson (2013)
	Washington (DC)	1	Avni and Fischler (2020)
	Medellín	1	Anguelovski et al. (2018)
	Total	7	

^a^Indicates papers where both greenways and other parks were explicitly considered.
